# Simplified end stage renal failure risk prediction model for the low-risk general population with chronic kidney disease

**DOI:** 10.1371/journal.pone.0212590

**Published:** 2019-02-22

**Authors:** Cynthia C. Lim, Miao Li Chee, Ching-Yu Cheng, Jia Liang Kwek, Majorie Foo, Tien Yin Wong, Charumathi Sabanayagam

**Affiliations:** 1 Department of Renal Medicine, Singapore General Hospital, Singapore, Singapore; 2 Singapore Eye Research Institute, Singapore National Eye Centre, Singapore, Singapore; 3 Ophthalmology and Visual Sciences Academic Clinical Program, Duke-NUS Medical School, Singapore, Singapore; 4 Department of Ophthalmology, Yong Loo Lin School of Medicine, National University of Singapore, Singapore, Singapore; Icahn School of Medicine at Mount Sinai, UNITED STATES

## Abstract

**Background:**

Chronic kidney disease (CKD) contributes significant morbidity and mortality among Asians; hence interventions should focus on those most at-risk of progression. However, current end stage renal failure (ESRF) risk stratification tools are complex and not validated in multi-ethnic Asians. We hence aimed to develop an ESRF risk prediction model by taking into account ethnic differences within a fairly homogenous socioeconomic setting and using parameters readily accessible to primary care clinicians managing the vast majority of patients with CKD.

**Methods:**

We performed a prospective cohort study of 1970 adults with CKD estimated glomerular filtration rate <60 ml/min/1.73m^2^ or albuminuria >30 mg/g from the population-based Singapore Epidemiology of Eye Diseases study (n = 10,033). Outcome was incident ESRF, ascertained by linkage to the Singapore Renal Registry until 2015.

**Results:**

Mean follow up was 8.5 ± 1.8 years and ESRF occurred in 32 individuals (1.6%). ESRF incidence rates were 2.8, 0.8 and 2.6 per 1000 patient years in Malays, Indians and Chinese respectively. The best ESRF prediction model included age, gender, eGFR and albuminuria (calibration χ2 = 0.45, P = 0.93; C-statistic 0.933, 95% confidence interval (CI) 0.889–0.978, p = 0.01; AIC 356). Addition of ethnicity improved discrimination marginally (C statistic 0.942, 95% CI 0.903–0.981, p = 0.21). Addition of clinical variables such as diabetes and hyperlipidemia did not improve model performance significantly.

**Conclusion:**

We affirmed the utility of commonly available clinical information (age, gender, eGFR and UACR) in prognosticating ESRF for multi-ethnic Asians with CKD.

## Introduction

Chronic kidney disease (CKD) increases risks of end-stage renal failure (ESRF) and cardiovascular disease [1] and ranked among the 10 most frequent causes of death and/or factors driving death and disability in India, Taiwan, China, Indonesia, Malaysia, Singapore and Australia according to the 2016 Global Burden of Disease Study [1, 2]. CKD is estimated to occur in 20–40% of Asian patients attending primary care clinics [3–5], thus placing a significant burden on primary healthcare and specialist nephrology services to evaluate and optimize control of risk factors to reduce progression to ESRF. However, renal function trajectories differ among patients with CKD and healthcare resources should be utilized effectively to focus CKD retardation efforts and more intensive monitoring in high-risk patients. There is thus an urgency to identify those most at-risk of progression to ESRF. The popular Kidney Failure Risk Equation (KFRE) developed in Canada incorporates demographic and biochemical parameters such as estimated glomerular filtration rate (eGFR), albuminuria, serum calcium, phosphate bicarbonate and albumin [6]. A multi-national meta-analysis with few Asian cohorts found that the model achieved good discrimination but over-estimated risk in non-North American groups [7]. Singapore has a multi-ethnic population with three major ethnic groups (Chinese, Malay and Indian) common in Asia thus enabling evaluation of ethnic differences within a fairly homogenous socioeconomic setting. As prevalence and risk factors of CKD appear to differ among the three ethnic groups [8], the broad application of a standard calibration factor for non-North American cohorts derived from the aforementioned meta-analysis may be inappropriate for the local CKD population [7]. Moreover, it is also uncertain if the slight improvement in discrimination and calibration justified the extra complexity of the 8-variable KFRE when compared with other simpler models [9]. Other renal failure prediction models used cystatin C or kidney histology [10–12], parameters not readily obtainable in primary care thus limiting their usefulness to clinicians managing the vast majority of patients with CKD. We aimed to develop and validate a risk prediction model for predicting the risk of ESRF using commonly available clinical variables easy to apply in the primary care setting, using data from the Singapore Epidemiology of Eye Diseases (SEED) study, a prospective, community-based cohort of Chinese, Malays and Indians in Singapore.

## Materials and methods

### Study population

SEED is a population-based cohort study of Chinese, Malay and Indian adults (n = 10,033) aged 40–80 years at baseline aimed to investigate the prevalence, incidence and risk factors of age-related eye diseases, and also the burden of major systemic diseases such as diabetes, hypertension and CKD. SEED included three independent population-based studies, the Singapore Malay Eye Study (SiMES, 2004–2006), the Singapore Indian Eye Study (SINDI, 2007–2009) and the Singapore Chinese Eye Study (SCES, 2009–2011) [13, 14]. Detailed methodology for these studies was previously reported [15]. Subjects were recruited in the same geographical area using age-stratified random sampling from computer-generated random lists of individuals 40 to 80 years of age residing in Singapore. All 3 studies followed similar protocols and were conducted in the same center (Singapore Eye Research Institute). The current study included 1970 persons who had CKD at baseline and outcome ESRD was obtained by linkage to the national renal registry. Previous investigators evaluating ESRF risk prediction used the Modification of Diet in Renal Diseases (MDRD) formula to estimate GFR [6, 9]. However, this formula tends to under-estimate GFR, possibly misclassifying patients with normal kidney function as mild CKD, while the Chronic Kidney Disease Epidemiology Collaboration (CKD-EPI) formula may be more accurate among those with normal or slightly lower GFR and correlated well with adverse outcomes internationally and among Asians [16–18]. Since our cohort was derived from population-based studies and thus more likely to include individuals with better renal function than cohorts selected from nephrology or hospital-based clinics, we considered the CKD-EPI equation to be more suitable to calculate eGFR for subjects in this study. CKD was defined according to modified Kidney Disease: Improving Global Outcomes (KDIGO) 2012 clinical practice guideline as eGFR <60 ml/min/1.73m^2^ or albuminuria (urinary albumin-to-creatinine ratio, UACR) >30 mg/g [19, 20]. Among 2524 individuals with eGFR <60 ml/min/1.73m^2^ or UACR >30 mg/g, we excluded those with Stage 5 CKD (eGFR <15 ml/min/1.73 m^2^, n = 22) or had missing data on serum creatinine (n = 22) or urinary albumin-to-creatinine ratio (n = 510). Thus, 1970 individuals were included in the development cohort.

### Data collection

An interviewer-administered questionnaire was used to collect participants’ socio-demographic, lifestyle and medical history. Smoking was classified into current smoker and former or non-smokers. Physical examination included weight, height and clinic blood pressure (BP). Body mass index (BMI) was calculated as weight in kilograms divided by the square of height in meters (kg/m^2^). Systolic BP and diastolic BP were measured using a digital automatic BP monitor (Dinamap model Pro Series DP110X-RW, 100V2; GE Medical Systems Information Technologies Inc., USA) after the participant was seated for at least 5 min. BP was measured twice, 5 minutes apart. A third measurement was made if the systolic BP differed by more than 10 mmHg or the diastolic BP by more than 5 mmHg. The mean between the two closest readings were then taken as the blood pressure for that individual. Hypertension was present if systolic BP was ≥140 mmHg or diastolic ≥90 mmHg or individuals reported previously physician-diagnosed hypertension or use of blood-pressure lowering medication. Diabetes mellitus was defined as random serum glucose ≥11.1 mmol/L or glycosylated haemoglobin (HbA1c) ≥6.5% or self-reported physician-diagnosed diabetes or use of glucose-lowering medication [21]. Hyperlipidemia was defined as total cholesterol ≥6.2 mmol/L or use of lipid lowering medication. Non-fasting venous blood was tested for serum lipids, glucose, HbA1c and creatinine. Serum creatinine was measured by enzymatic method (SiMES) or Jaffe method (SINDI and SCES) calibrated to the National Institute of Standards and Technology (NIST) Liquid Chromatography Isotope Dilution Mass Spectrometry (LC-IDMS) and expressed in micromoles per liter (μmol/L). A single random spot urine sample was used to measure urine albumin to creatinine ratio (UACR, mg/g) using commercial assay (Immulite, DPC, United Kingdom). The lower detection limits for urinary albumin and creatinine were 0.5 mg/L and 0.027 mmol/L respectively. UACR was available only in a third of the Malay participants (those with known diabetes and 1 in 5 with no diabetes). Consequently, 473 of 719 participants from the SiMES cohort with eGFR < 60 ml/min/1.73 m^2^ were excluded due to missing UACR values. All laboratory investigations were conducted at the National University Hospital Reference Laboratory (SiMES) and Singapore General Hospital (SINDI, SCES) which are accredited by the College of American Pathologists.

Written informed consent was obtained from all participants before enrolment. This study was approved by the Singapore Eye Research Institute Review Board and conduct of the study adhered to the Declaration of Helsinki.

### Outcome definition

Primary outcome of interest in this study was incident ESRF, defined as eGFR less than 15 ml/min/1.73 m^2^, serum creatinine more than 500 μmol/L or 5.7 mg/dL, or if subject received transplantation or chronic dialysis. ESRF was ascertained by linking the study cohort to the Singapore Renal Registry at National Registry of Diseases Office until 2015. The Singapore Renal Registry collates voluntary submissions of new ESRF patients from all public and private centers in Singapore with estimated data capture of 95% of all dialysis patients [22].

### Statistical analysis

All statistical analyses were performed using STATA statistical software (Version 14.1, StataCorp, College Station, Texas). Baseline characteristics of the 3 ethnic groups with CKD were compared using analysis of variance (ANOVA) or chi-square test as appropriate for the variable. Uni-variate Cox proportional-hazards regression was performed to examine the association between demographic and clinical characteristics with incident ESRF. Variables associated with ESRF (P < 0.05) in uni-variate analysis and established clinical factors from literature (age, gender, eGFR, UACR, ethnicity, diabetes mellitus, hypertension and hyperlipidemia) were subsequently included in a series of multi-variable models for further analysis. Calibration was assessed using Nam-D’Agostino χ^2^ statistic to examine how closely each model’s predicted probabilities agreed with actual outcome, where χ^2^ values >20 with P<0.05 suggest poor agreement between predicted and observed outcomes [23]. Model comparison was done using Harrell’s Concordance statistic (C statistic) and Akaike Information Criterion (AIC). C statistic, the equivalent of the area (AUC) under a receiver operating characteristic (ROC) curve for a binary outcome variable [24], was computed as a measure of discrimination for ESRF in the developed models. AIC was computed to compare the goodness of fit between the various models, accounting for model complexity; difference in AIC >10 is considered significant [25]. Bootstrap sampling with 10,000 replications was then performed for the chosen prediction model to compute the standard errors and 95% CIs to check for robustness of the model coefficients. Lastly, we performed a sensitivity analysis for our best developed model among subjects with CKD stages 3–4.

## Results

We identified 1970 participants (382 Malays, 816 Indians and 772 Chinese) to have CKD at baseline (Table 1). Subjects’ mean age was 62.4 ± 10.2 years and half were female. Malay subjects in SiMES were more likely to be hypertensive with higher BMI, BP and cholesterol levels but lower eGFR. Indian participants in SINDI were younger, more likely to have diabetes and had higher glucose and HbA1c levels. Chinese participants in SCES were older but had better renal function. S1 Fig illustrates the distribution of the development cohort categorized in an eGFR and albuminuria grid, where lower eGFR and higher UACR incrementally increase risk of progressive CKD [19]. More than half the cohort (61.3%) had normal or mildly decreased eGFR (eGFR ≥60 ml/min/1.73 m^2^) with moderately increased albuminuria (30–300 mg/g) and thus at moderately increased risk of progressive CKD. In contrast, few (9.3%) had eGFR <45 ml/min/1.73 m^2^ and were at high to very high risk of progressive CKD.

**Table 1:** Baseline characteristics of subjects with chronic kidney disease categorized by ethnicity.

	ALL CKD	SiMES	SINDI	SCES	P-value
	N = 1970	n = 382	n = 816	n = 772	
Age, years	62.4 (10.2)	62.5 (10.4)	60.9 (10.0)	63.8 (10.2)	< 0.001
Female gender, n (%)	1050 (53.3)	216 (56.5)	421 (51.6)	413 (53.50)	0.28
Current smoker, n (%)	237 (12.0)	64 (16.7)	92 (11.2)	81 (10.4)	0.006
Diabetes mellitus, n (%)	882 (45.3)	164 (43.1)	478 (59.6)	240 (31.3)	< 0.001
Hyperlipidemia, n (%)	1092 (56.2)	201 (52.6)	465 (58.7)	426 (55.4)	0.11
Hypertension, n (%)	1534 (78.0)	328 (86.3)	601 (73.8)	605 (78.3)	< 0.001
Systolic BP, mmHg	146 (22)	159 (23)	143 (21)	144 (21)	< 0.001
Diastolic BP, mmHg	79 (11)	83 (12)	78 (11)	78 (9)	< 0.001
Total cholesterol, mmol/L	5.32 (1.20)	5.71 (1.25)	5.10 (1.18)	5.36 (1.13)	< 0.001
LDL cholesterol, mmol/L	3.21 (0.97)	3.35 (0.98)	3.19 (0.99)	3.15 (0.94)	0.005
HDL cholesterol, mmol/L	1.22 (0.38)	1.33 (0.34)	1.10 (0.34)	1.30 (0.40)	< 0.001
Body mass index, kg/m^2^	25.5 (4.8)	26.6 (5.0)	26.4 (5.2)	24.0 (3.7)	< 0.001
eGFR, ml/min/1.73 m^2^	75.97 (24.17)	58.39 (15.67)	80.13 (23.02)	80.29 (24.89)	< 0.001
UACR, mg/g	158.88 (484.50)	205.55 (652.21)	148.89 (447.95)	146.35 (419.33)	0.11
Serum glucose, mmol/L	7.8 (4.4)	7.9 (4.5)	8.4 (4.6)	7.2 (4.1)	< 0.001
HbA1c, %	6.7 (1.59)	6.8 (1.8)	6.9 (1.7)	6.3 (1.2)	< 0.001
Time to ESRD, years	4.4 (2.3)	6.0 (2.3)	3.6 (2.6)	3.5 (1.6)	0.01
Follow-up, years	8.5 (1.8)	11.1 (1.0)	8.9 (0.7)	6.7 (0.9)	< 0.001

Abbreviations: BP, blood pressure; CKD, chronic kidney disease; eGFR, estimated glomerular filtration rate; ESRD, end stage renal disease; HbA1c, glycosylated hemoglobin A1; HDL, high density lipoprotein; LDL, low density lipoprotein; SCES, Singapore Chinese Eye Study; SiMES, Singapore Malay Eye Study; SINDI, Singapore Indian Eye Study; UACR, urine albumin to creatinine ratio; Values for categorical variables are reported as number (percentage) and continuous variables reported as mean (standard deviation).

Total follow up was 8.5 ± 1.8 years and ESRF occurred in 32 subjects (1.6%) at 4.4 ± 2.3 years. S2 Fig shows incident ESRF categorized by ethnicity. Incidence rates of ESRF were 2.8, 0.8 and 2.6 per 1000 patient years respectively in Malay, Indian and Chinese subgroups. Among those who had ESRF, fewer than half (13 subjects, 40.6%) had early ESRF within 5 years (8, 3 and 2 subjects from SiMES, SINDI and SCES respectively). Table 2 shows the uni-variate analysis for factors associated with ESRF. Indians, compared to Chinese, were less likely to develop ESRF despite a significantly longer follow-up duration (11.1±1.0 versus 6.8±0.9 years, p<0.001). Diabetes, hyperlipidemia, lower eGFR and higher albuminuria, serum glucose and HbA1c were significantly associated with ESRF.

**Table 2:** Hazard ratios, calibration and goodness of fit for models for end stage renal failure.

Variable	Model 1			Model 2			Model 3			Model 4			Model 5		
	HR	95% CI	p-value	HR	95% CI	p-value	HR	95% CI	p-value	HR	95% CI	p-value	HR	95% CI	p-value
Age per 10 years	0.48	0.32, 0.72	< 0.001	0.69	0.46, 1.03	0.07	0.62	0.41, 0.93	0.021	0.68	0.45, 1.02	0.06	0.67	0.44, 1.02	0.06
Female	1.06	0.53, 2.12	0.87	0.88	0.44, 1.76	0.71	0.80	0.38, 1.67	0.55	1.06	0.52, 2.16	0.88	1.18	0.57, 2.43	0.66
Race															
Chinese								Reference							
Malay							0.53	0.23, 1.23	0.14						
Indian							0.28	0.10, 0.78	0.015						
eGFR per 5 ml/min/1.73 m^2^	0.59	0.53, 0.67	< 0.001	0.68	0.61, 0.77	< 0.001	0.69	0.62, 0.77	< 0.001	0.69	0.62, 0.77	< 0.001	0.70	0.62, 0.78	< 0.001
Log Albuminuria				1.71	1.38, 2.12	< 0.001	1.70	1.37, 2.11	< 0.001	1.67	1.33, 2.09	< 0.001	1.61	1.29, 2.00	< 0.001
Diabetes										2.65	0.99, 7.13	0.05	2.60	0.97, 6.97	0.06
Hypertension										0.66	0.19, 2.34	0.52			
Hyperlipidemia													2.09	0.79, 5.49	0.14
C Statistic	0.89	0.82, 0.95		0.93	0.889, 0.978	0.010^a^	0.942	0.903, 0.981	0.21^a^	0.946	0.914, 0.977	0.07^a^	0.939	0.899, 0.980	0.13^a^
Akaike Information Criterion	379			356			353			355			345		
Nam-D’Agostino χ^2^	26.43		< 0.001	0.45		0.93	0.44		0.93	1.60		0.66	0.46		0.93

Model 1: age, gender, eGFR. Model 2: age, gender, eGFR, albuminuria. Model 3: age, gender, race, eGFR, albuminuria. Model 4: age, gender, eGFR, albuminuria, diabetes, hypertension. Model 5: age, gender, eGFR, albuminuria, diabetes, hyperlipidaemia

^a^P values are for comparison of C statistics between successive models for model 1–3. Models 4 and 5 were compared with model 2.

eGFR, estimated glomerular filtration rate

**Fig 1:**
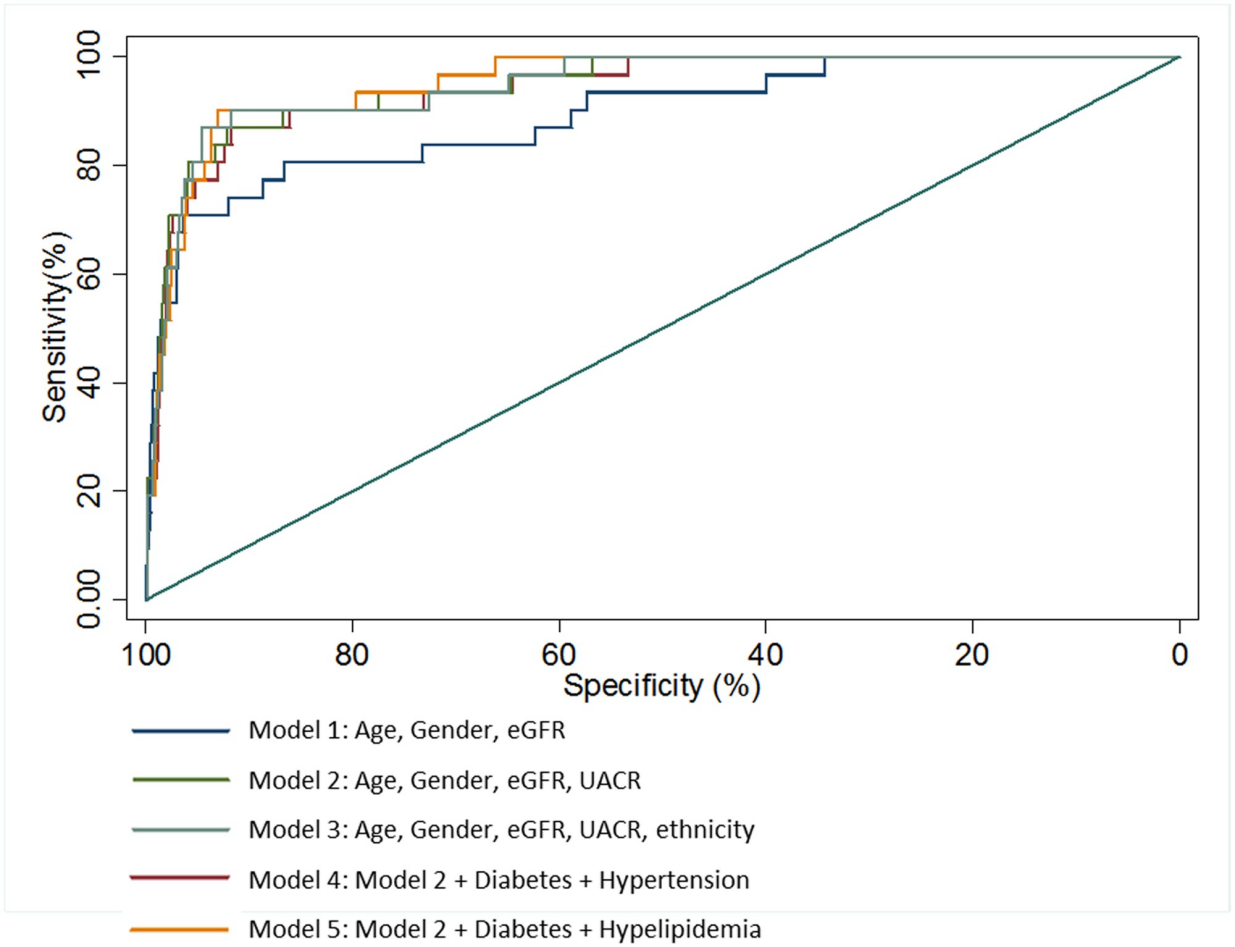
Receiver operating characteristic (ROC) curves for models 1–5 for discriminating persons with and without incident end-stage renal failure (ESRF). Area under the curve (AUC) and 95% confidence intervals (CI) for Models 1 (AUC 0.933, 95% CI 0.889–0.978, p = 0.01) was significantly better compared to Model 1 (AUC 0.885, 95% CI 0.816–0.953). Discrimination by Models 3 (AUC 0.942, 95% CI 0.903–0.981, p = 0.21), 4 (AUC 0.946, 95% CI 0.914–0.977, p = 0.07) and 5 (AUC 0.939, 95% CI 0.899–0.980, p = 0.13) were not significantly different compared with Model 2. The straight line representing an AUC of 0.5 indicates inability to differentiate between outcomes, whereas the ideal predictive model should have an AUC of 1.0.

Table 2 shows hazard ratios for the variables and C-statistics and AIC for successive models for ESRF. As it was well-established that baseline renal function measured by eGFR was associated with ESRF risk [19], and confirmed in our cohort (S1 Table), age, gender and eGFR were included in Model 1 and performed fairly well in both discrimination and goodness of fit. However, the Nam–D’Agostino chi-squared test for model 1 indicated inadequate calibration (P<0.001). Addition of albuminuria in Model 2 improved calibration (χ^2^ = 0.45, P = 0.93), C-statistic (0.933, 95% confidence interval (CI) 0.889–0.978, p = 0.01) and AIC. Further addition of ethnicity in Model 3 improved the C-statistic marginally (0.942, 95% CI 0.903–0.981) but this was not statistically significant. Addition of other commonly available co-morbidity information (diabetes and hypertension in Model 4; diabetes and hyperlipidemia in Model 5) did not significantly alter discrimination and goodness of fit compared to Model 2. Again, addition of ethnicity to Models 4 and 5 did not significantly improve model performance (C-statistic 0.954, 95% CI 0.925–0.982, p = 0.22 and C statistic 0.948, 95% CI 0.911–0.985, p = 0.10 respectively, not presented). Models 3 to 5 all had adequate calibration (all P>0.60). Fig 1 demonstrates the ROC curves for Models 1–5 in predicting ESRF. AUC for Model 1 was ≥0.80, suggesting excellent discriminatory power, while Models 2 to 5 had AUC ≥0.90, suggesting outstanding discriminatory power. Model 2 had significantly better discrimination (p = 0.01) than Model 1 but AUC for Models 3, 4 and 5 were not significantly different compared to Model 2. Sensitivity analysis in subjects with CKD Stage 3 and 4, i.e. those with eGFR 15 to 60 ml/min/1.73 m^2^, confirmed consistently high discriminatory value and goodness of fit with Model 2 (C-statistic 0.942, AIC 232).

We performed internal validation of our model by using bootstrap sampling with 10,000 replications and computed the standard errors and 95% confidence intervals (CIs) of the estimated coefficients to check for robustness of our prediction model (Model 2) coefficients. The bootstrapped results were generally consistent with our original model, except that age which was marginally significantly associated with ESRF (P = 0.07) in our original model was shown to be associated with ESRF based on bootstrap results (P = 0.042). In addition, the bootstrapped standard errors were reasonably similar and do not differ much from the original model, of which the bootstrapped confidence intervals were close to our original CIs (S2 Table). Hence the results suggest that there is little, if any, bias in the coefficients obtained and used for prediction for final chosen model 2.

As the variables in Model 2 were consistent with the 4-variable KFRE in direction but differed in coefficient magnitude (Table 3), we compared calibration of Model 2 with the 4-variable KFRE with and without regional calibration factor (S3 Table) using the Brier score, an aggregate measure of disagreement between observed outcome and its prediction. The score is derived by taking the mean squared difference between predicted risk probability and actual outcome and ranges from 0 to 1, where 0 indicates perfect predictions and a value of 0.33 or greater indicating random predictive ability. The lower the Brier score is for a set of predictions, the better the predictions are calibrated. Brier scores for Model 2, the 4-variable KFRE and the calibrated 4-variable KFRE were 0.0131, 0.0124 and 0.0125 respectively. Comparison of Brier scores using Wilcoxon sign rank test found that the 4-variable KFRE and calibrated 4-variable KFRE had significantly better calibration than Model 2 (p<0.001). We further confirmed that the 4-variable KFRE model demonstrated good discrimination in our study cohort [C Statistic = 0.91; 95% CI (0.86–0.97)].

**Table 3:** Hazard ratios and beta coefficients for Model 2 and 4-variable Kidney Failure Risk Equation.

	Model 2		4-variable KFRE	
	HR	Beta	HR	Beta
Male	1.14	0.13	1.26	0.27
Age per 10yr	0.69	-0.37	0.80	-0.22
eGFR per 5 mL/min/1.73 m^2^	0.68	-0.38	0.57	-0.55
Log Albuminuria	1.71	0.54	1.60	0.46

Abbreviations: eGFR, estimated glomerular filtration rate; KFRE, Kidney Failure Risk Equation

## Discussion

We evaluated ESRF risk prediction models for multi-ethnic Asians with CKD using readily available demographic and co-morbidity data and laboratory values for tests commonly performed in primary care [3]. The high C-statistics of 0.933 (95% CI 0.889–0.978) and 0.942 (95% CI 0.903–0.981) for Model 2 (age, gender, eGFR and UACR) and 3 (age, gender, ethnicity, eGFR and UACR) respectively, confirmed excellent discrimination i.e. the ability to differentiate subjects who developed ESRF from those who did not. The discrimination afforded by our models was similar to that achieved by the 4-variable KFRE for predicting ESRF [7], despite lower incidence of ESRF (1.9 per 1000 patient years) in our cohort compared with other studies which reported kidney failure incidence between 10 and 80 per 1000 patients years [7]. Notably, our cohort was derived from population-based studies and had less severe renal dysfunction with higher baseline eGFR (mean 75.9 ±24.1 ml/min/1.73 m^2^) compared with studies which included subjects with CKD stage 3–5 recruited from hospital-based diabetic or nephrology clinics and hence at higher risk for progressive CKD [6, 26]. We addressed this in the sensitivity analysis which showed that our developed Model 2 performed well among individuals with CKD Stage 3 and 4 with more severe renal dysfunction.

In addition, we confirmed that the ethnicity altered ESRF risks but its’ addition to the highly-discriminating Model 2 (age, gender, eGFR and UACR) did not significantly improve model performance (p = 0.21). This study affirmed the utility of age, gender, eGFR and UACR in prognosticating ESRF, as evaluated by the 4-variable KFRE^7^. Although a calibration factor for non-North American cohorts was added to the KFRE (S3 Table), this was largely based on European or East Asian populations and thus limited its generalizability to the multi-ethnic Southeast Asian population. In this study, calibration of the 4-variable KFRE was not altered by addition of the regional calibration factor (Brier scores 0.0124 and 0.0125 respectively).

Use of these predictive models to estimate individualized risk of progression to ESRF can improve planning for frequency of follow up and engaging patients in shared decision making, especially since certain treatment options such as referral to nephrology services at tertiary care centers and advance care or dialysis planning involve significant healthcare costs and risks and should be offered to those most at-risk of ESRF. Table 4 demonstrates use of Model 2 and the 4-variable KFRE in estimating absolute risk of ESRF at 5 years. Compared with 4-variable KFRE, model 2 of the current study increases the predicted risk by 1.3% and 0.6% for patients A and B at low risk for ESRF, but decreased the risk by 1.3% and 2.7% in patients C and D at greater risk for ESRF. Although the absolute risk difference is not large, Model 2 tends to predict a lower risk of ESRF among those with more severe renal impairment, possibly because our development cohort had milder CKD compared to the KFRE development cohort from CKD clinics [6].

**Table 4:** Predicted probability of end stage renal failure at 5 years for 4 Hypothetical Patient Profiles based on Model 2 and the 4-variable Kidney Failure Risk Equation.

	Patient A 50 year old female, eGFR 50 ml/min/1.73 m^2^ and UACR 100 mg/g	Patient B 60 year old male, eGFR 50 ml/min/1.73 m^2^ and UACR 100 mg/g	Patient C 50 year old female, eGFR 30 ml/min/1.73 m^2^ and UACR 100 mg/g	Patient D 60 year old female, eGFR 30 ml/min/1.73 m^2^ and UACR 300 mg/g
Model 2	2.9	2.3	12.8	15.6
4-variable KFRE	1.6	1.7	14.1	18.3

Abbreviations: eGFR, estimated glomerular filtration rate; KFRE, Kidney Failure Risk Equation. Predicted probabilities calculated according the risk equations for Model 2 (Risk = 1–0.998^exp (-0.382*[(eGFR/5)-15.19]+0.133*(male-0.467) + 0.536*[ln(UACR)-4.016] -0.374*[(age/10)-6.24]) and the 4-variable KFRE (Risk = 1–0.924^exp(-0.554*[(eGFR/5)-7.22]+0.269*(male-0.560) + 0.456*[ln(UACR)-5.277] -0.217*[(age/10)-7.04]) and presented as percentages.

An alternate prediction model validated locally for risk of CKD progression, defined as worsening of eGFR categories or ≥25% reduction in eGFR from baseline, is limited to diabetics [24]. Among 1582 local diabetic patients followed up for median 5.5 years by a hospital-practice diabetic center, approximately 40% had progressive CKD but inclusion of milder forms of CKD progression, such as from CKD Stage 1 to 2, in the outcome definition makes the alternate prediction model less useful for the purpose of identifying patients most at-need of intensive healthcare resources such as nephrology referrals and dialysis planning.

Strengths of this study include the inclusion of a large, well-characterized multi-ethnic Asian sample, a complete and robust data set and long follow up. However, lack of UACR values in two-thirds of Malay participants in SiMES resulted in excluding a large portion of Malay participants with eGFR <60 ml/min/1.73 m^2^ from this study. Notably, prevalence of CKD (defined as eGFR <60 or UACR >30 mg/g) was highest among Malays (44.7% versus 27.6% and 25.5% among Indians and Chinese respectively). Selection bias may result if those with missing UACR values had lower or greater risk for ESRF outcome than those who were included in the cohort. Our assessment of diabetes based on a single measure of random blood glucose ≥11.1 mmol/L may have resulted in nondifferential misclassification of diabetes status which is a limitation inherent to large population-based epidemiological studies. The low incidence of ESRF limited the number of variables in the model that could be adjusted for and limited power to detect effect of ethnicity on ESRF risk. We were unable to directly compare our model with the 8-variable KFRE since our dataset did not include biochemistry values for serum calcium, phosphate, albumin and bicarbonate but the discriminatory values of both models were similar. Multiple large cross-sectional studies have shown that serum calcium and phosphate are unlikely to be markedly deranged before CKD Stage 3b (eGFR <45 ml/min/1.73 m^2^) [27, 28]. The United States Third National Health and Nutrition Examination Survey (NHANES III) studied data from 15,594 subjects and found that serum albumin was <38 g/L in 10% of those with eGFR 60–89 ml/min/1.73 m^2^ compared to 20% and 51% among those with CKD stage 3 and 4 respectively (P<0.001) [29]. Similarly, serum bicarbonate was <22 mmol/L in 1% of those with eGFR 60–89 ml/min/1.73 m^2^ compared to 2% and 19% among those with CKD stage 3 and 4 respectively (P<0.001) [29]. Hence we postulate that addition of these variables in prediction models, such as the 8-variable KFRE, is unlikely to markedly alter ESRF risk possibilities in subjects with mild CKD such as our cohort. CKD is under-documented in local primary care clinics and screening for chronic kidney disease among at-risk individuals remains suboptimal in many countries [3, 30, 31]. Hence, additional biochemistry results (serum calcium, phosphate, bicarbonate, albumin) required by the 8-variable KFRE may not be readily available and may increases both patients’ out-of-pocket expenditure and healthcare costs. Instead, a low-cost model that uses commonly available clinical data to accurately predict ESRF in the general population with CKD will be a more useful clinical decision support tool for the primary healthcare practitioner to guide frequency of follow up with renal function tests or need for nephrology referral.

## Conclusion

We affirmed the utility of clinical information that are commonly available in the primary care setting (age, gender, eGFR and UACR) in prognosticating ESRF for multi-ethnic Asians with CKD.

## Supporting information

S1 Fig Baseline renal function of development cohort categorized by CKD stage according to eGFR and albuminuria criteria. (DOCX)

S2 Fig Incidence of end stage renal failure (ESRF) categorized by ethnicity. ESRF occurred in 12 Malays.
(DOCX)

S1 Table Association of demographic and clinical factors with end stage renal failure. (DOCX)

S2 Table Comparison of original and bootstrap results for Model 2 (based on 10,000 replications). (DOCX)

S3 Table Equations for 5-year risk of end stage renal failure according to Model 2 and 4-variable Kidney Failure Risk Equation without and with non-North American calibration factor. (DOCX)
