# Supplementary material for: Simplified end stage renal failure risk prediction model for the low-risk general population with chronic kidney disease
Source: PLoS One. 2019 Feb 22;14(2):e0212590. doi: 10.1371/journal.pone.0212590 (PMC6386264; doi:10.1371/journal.pone.0212590)
Supplement: S1 Fig — (DOCX) [file pone.0212590.s001.docx]

**S1 Fig. Baseline renal function of development cohort categorized by CKD stage according to eGFR and albuminuria criteria.**


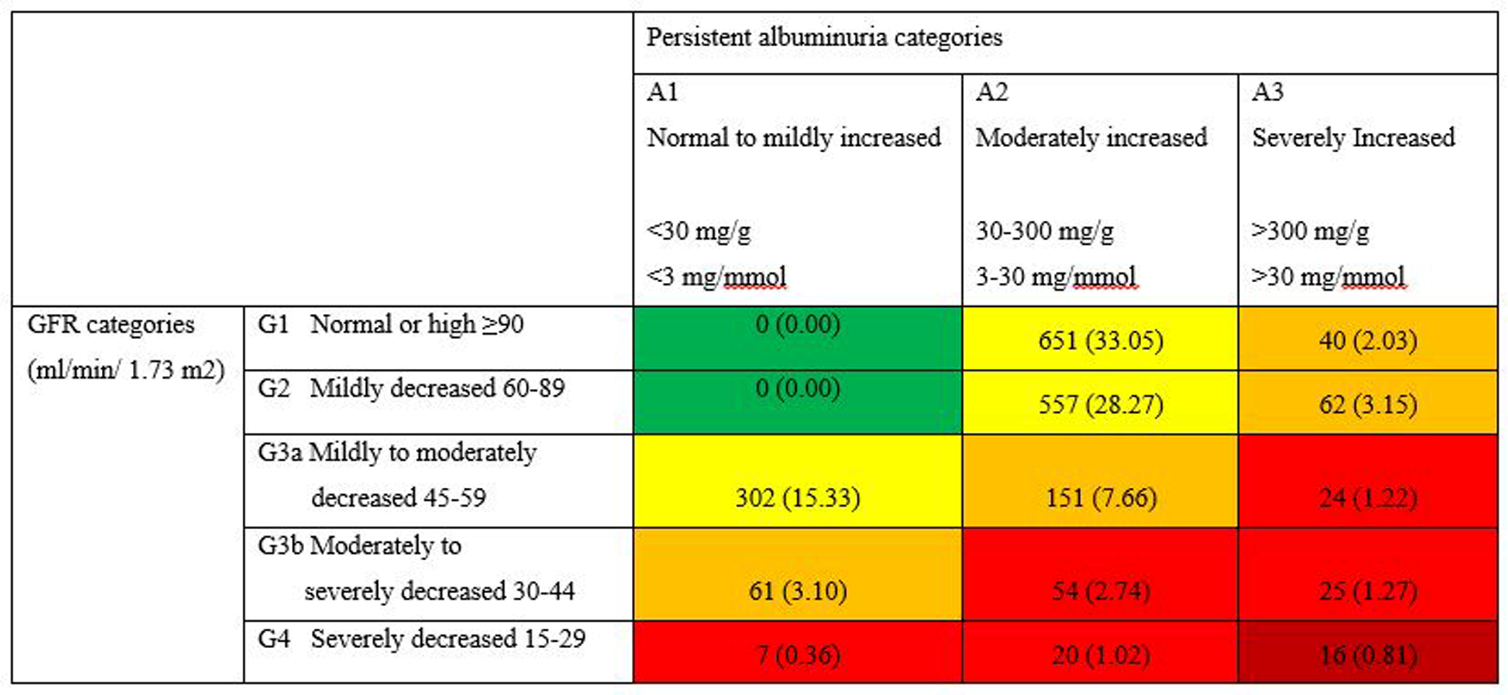

GFR and albuminuria grid reflects risk of progressive chronic kidney disease by colour. Green: low risk in absence of other markers of kidney disease; Yellow: moderately increased risk; Orange: high risk; Red very high risk. Numbers in boxes represent frequency and percentage.

**Supplementary Figure 2.** Incidence of End Stage Renal Failure (ESRF) categorized by ethnicity. ESRF occurred in 12 Malays

**
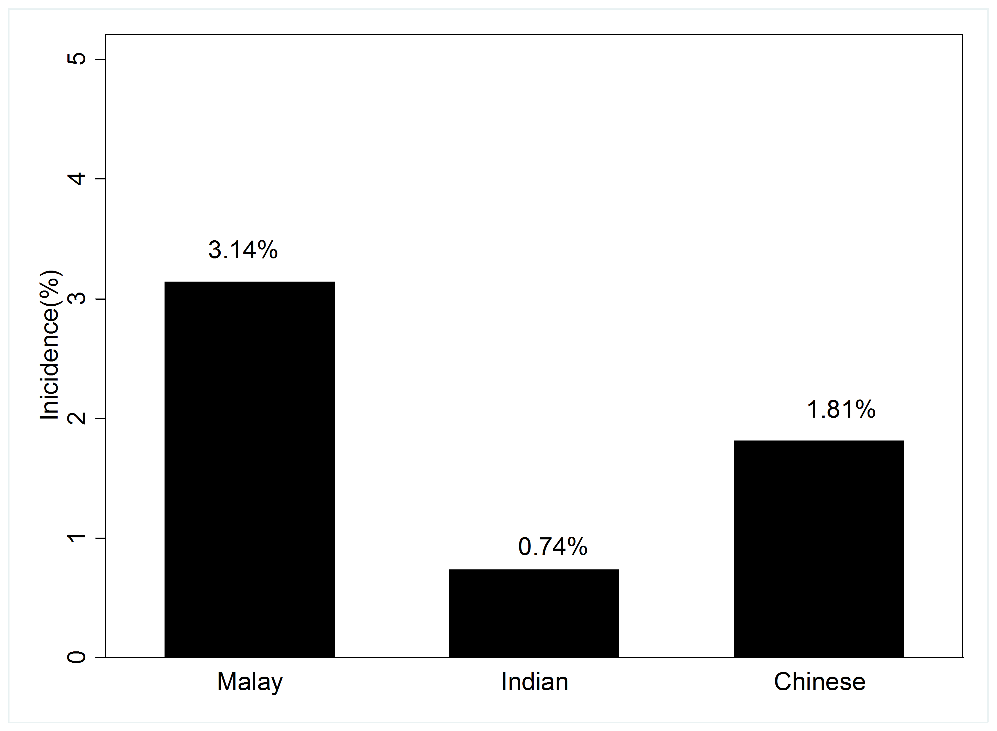
**

ESRF occurred in 12 Malays (3.14% of 382), 6 Indians (0.74% of 816) and 14 Chinese (1.81% of 772) with baseline chronic kidney disease, defined as estimated glomerular filtration rate less than 60 ml/min/1.73 m^2^ or urine albumin-to-creatinine ratio more or equal to 30 mg/g.
