# Supplementary material for: Simplified end stage renal failure risk prediction model for the low-risk general population with chronic kidney disease
Source: PLoS One. 2019 Feb 22;14(2):e0212590. doi: 10.1371/journal.pone.0212590 (PMC6386264; doi:10.1371/journal.pone.0212590)
Supplement: S2 Fig — ESRF occurred in 12 Malays. (DOCX) [file pone.0212590.s002.docx]

**S2 Fig.** Incidence of End Stage Renal Failure (ESRF) categorized by ethnicity. ESRF occurred in 12 Malays

**
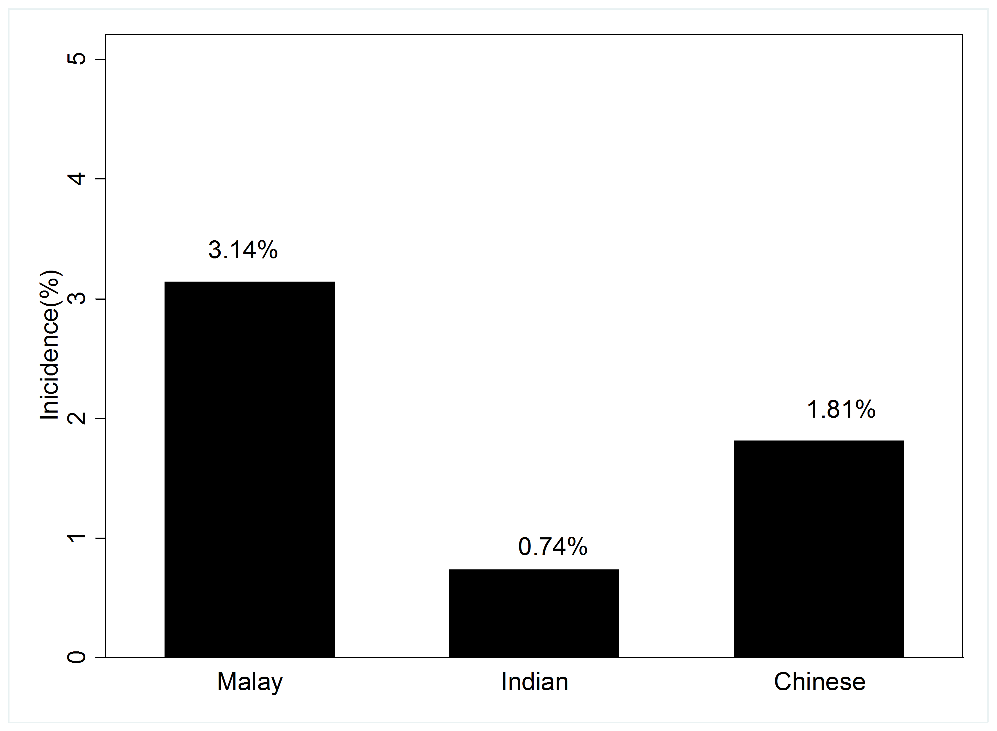
**

ESRF occurred in 12 Malays (3.14% of 382), 6 Indians (0.74% of 816) and 14 Chinese (1.81% of 772) with baseline chronic kidney disease, defined as estimated glomerular filtration rate less than 60 ml/min/1.73 m^2^ or urine albumin-to-creatinine ratio more or equal to 30 mg/g.
