# Supplementary material for: Simplified end stage renal failure risk prediction model for the low-risk general population with chronic kidney disease
Source: PLoS One. 2019 Feb 22;14(2):e0212590. doi: 10.1371/journal.pone.0212590 (PMC6386264; doi:10.1371/journal.pone.0212590)
Supplement: S1 Table — (DOCX) [file pone.0212590.s003.docx]

| **S1 Table. Association of demographic and clinical factors with end stage renal failure** | | | |
| --- | --- | --- | --- |
|  | Uni-variable model | | |
| Characteristics | HR | 95% CI | P-value |
| Age per 10 years | 1.07 | 0.77, 1.51 | 0.68 |
| Female gender, yes | 0.77 | 0.39, 1.54 | 0.46 |
| Ethnicity, yes |  |  |  |
| Chinese | Reference | | |
| Malay | 1.21 | 0.54, 2.70 | 0.64 |
| Indian | 0.29 | 0.11, 0.76 | 0.012 |
| Current smoker, yes | 1.02 | 0.36, 2.91 | 0.97 |
| Diabetes mellitus, yes | 6.18 | 2.38, 16.06 | < 0.001 |
| Hyperlipidemia, yes | 4.09 | 1.57, 10.65 | 0.004 |
| Hypertension, yes | 2.76 | 0.84, 9.05 | 0.09 |
| Systolic BP, per 1 mmHg | 1.01 | 0.99, 1.02 | 0.33 |
| Diastolic BP, per 1 mmHg | 0.99 | 0.96, 1.02 | 0.43 |
| Total cholesterol, per 1 mmol/L | 1.19 | 0.91, 1.56 | 0.21 |
| LDL cholesterol, per 1 mmol/L | 0.81 | 0.56, 1.19 | 0.29 |
| HDL cholesterol, per 1 mmol/L | 0.46 | 0.16, 1.31 | 0.14 |
| Body mass index, per 1 kg/m^2^ | 1.04 | 0.98, 1.11 | 0.15 |
| eGFR, per 5 ml/min/1.73 m^2^ | 0.62 | 0.55, 0.70 | < 0.001 |
| Log UACR | 2.51 | 2.04, 3.09 | < 0.001 |
| Serum glucose (per 1 mmol/L) | 1.08 | 1.05, 1.12 | < 0.001 |
| HbA1c (per 1 % increase) | 1.36 | 1.19, 1.56 | < 0.001 |
| Abbreviations : BP, blood pressure; CKD, chronic kidney disease; eGFR, estimated glomerular filtration rate; HbA1c, glycosylated hemoglobin A1; HDL, high density lipoprotein; LDL, low density lipoprotein; UACR, urine albumin to creatinine ratio; | | | |
