# Supplementary material for: Simplified end stage renal failure risk prediction model for the low-risk general population with chronic kidney disease
Source: PLoS One. 2019 Feb 22;14(2):e0212590. doi: 10.1371/journal.pone.0212590 (PMC6386264; doi:10.1371/journal.pone.0212590)
Supplement: S2 Table — (DOCX) [file pone.0212590.s004.docx]

| **S2 Table. Comparison of original and bootstrap results for Model 2 (based on 10,000 replications)** | | | | |
| --- | --- | --- | --- | --- |
|  | **Original** | | **Bootstrap** | |
|  | **SE** | **(95% CI)** | **SE** | **(95% CI)** |
| Age per 10 years | 0.14 | (0.46, 1.03) | 0.13 | (0.48, 0.99) |
| Female | 0.31 | (0.44, 1.76) | 0.37 | (0.39, 1.99) |
| eGFR per 5 ml/min/1.73 m^2^ | 0.04 | (0.61, 0.77) | 0.06 | (0.57, 0.82) |
| Log Albuminuria | 0.19 | (1.38, 2.12) | 0.23 | (1.31, 2.22) |
| Abbreviations: eGFR, estimated glomerular filtration rate | | | | |
