# Supplementary material for: Simplified end stage renal failure risk prediction model for the low-risk general population with chronic kidney disease
Source: PLoS One. 2019 Feb 22;14(2):e0212590. doi: 10.1371/journal.pone.0212590 (PMC6386264; doi:10.1371/journal.pone.0212590)
Supplement: S3 Table — (DOCX) [file pone.0212590.s005.docx]

| **S3 Table. Equations for 5-year risk of End Stage Renal Failure according to Model 2 and 4-variable Kidney Failure Risk Equation without and with non-North American calibration factor** | |
| --- | --- |
| Model 2 | 1 – 0.998^exp (-0.382*[(eGFR/5)-15.19]+0.133*(male-0.467) + 0.536*[ln(UACR)-4.016] -0.374*[(age/10)-6.24]) |
| 4-variable KFRE | 1 - 0.924^exp(-0.554*[(eGFR/5)-7.22]+0.269*(male-0.560) + 0.456*[ln(UACR)-5.277] -0.217*[(age/10)-7.04]) |
| Calibrated 4-variable KFRE | 1 - 0.936^exp(-0.554*[(eGFR/5)-7.22]+0.269*(male-0.560) + 0.456*[ln(UACR)-5.277] -0.217*[(age/10)-7.04]) |
| Where 5-year risk = 1- ${S_{0}(t)}^{\exp(f\left( x \right))}$ and $f\left( x \right)= \beta_{1}(x_{1}-\bar{x_{1})}+\beta_{2}(x_{2}-\bar{x_{2})}+\ldots.+\beta_{p}(x_{p}-\bar{x_{p})}$  $S_{0}(t=5)$ represents the survival probability of end stage renal failure at 5 years and $\beta_{1},\ldots.,\beta_{p}$ represents the regression coefficients from cox proportional hazrd regression model.  Abbreviations : eGFR, estimated glomerular filtration rate; UACR, urine albumin to creatinine ratio; KFRE, Kidney Failure Risk Equation | |
